# Antioxidant Activity of Different Extracts from Black Alder (*Alnus glutinosa*) Bark with Greener Extraction Alternative

**DOI:** 10.3390/plants10112531

**Published:** 2021-11-21

**Authors:** Maris Lauberts, Matiss Pals

**Affiliations:** Latvian State Institute of Wood Chemistry, Dzerbenes Street 27, LV-1006 Riga, Latvia; matiss232@gmail.com

**Keywords:** extraction, green solvent, organic solvents, total phenolic content, antioxidant activity

## Abstract

Phenolic compounds isolated from plant biomass consist of bioactive components showing a wide range of benefits for humans, including antioxidant, antimicrobial or anti-inflammatory effects. This paper presents the potential value of black alder (*Alnus glutinosa* (L.) Gaertn. (*Betulaceae*)) bark for the production of biologically active substances, despite its current use as a low value fuel source. Most of the extraction methods employ neat organic solvents to obtain extracts with a high antioxidant potential from biomass. The aim of this work is to show the advantages and disadvantages of the extraction process by taking into account the principles of ‘green chemistry’ and replacing the organic solvents with ‘green’ solvent water. Using the advantages of accelerated solvent extraction (ASE), it has been shown that the use of deionized water has the prospect of replacing organic solvents. In the case of the one-step water extraction, the total polyphenol content (TPC) varies from 0.55 to 0.62 Gallic acid equivalent (GAE) g/g in the extracts, depending on the temperature, whereas with the result of the sequential extraction with the organic solvents, the TPC content of the 40% (v:v) ethanol extracts ranges from 0.39 to 0.61 GAE g/g, depending on the temperature. The influence of the total polyphenol content and the total proanthocyanidin content on the antioxidant activity is shown. The antioxidant activity (IC_50_, mg/L) of the extracts obtained with the organic solvents in the (2,2-diphenyl-1-picrylhydrazyl) DPPH^•^ test varies from 4.05 to 9.58, depending on the temperature in the range of 70–150 °C, respectively, while the results obtained with the deionized water showed promising results in the range of 6.33–7.36 in the temperature range of 70–150 °C, respectively. The extraction with the deionized water showed that approximately 90% of the substances in the extracts obtained with the organic solvents by sequential extraction are possible to obtain as deionized water extracts.

## 1. Introduction

The genus *Alnus*, also known as alder, belonging to the birch (*Betulaceae*) family is a tree species that grows throughout the northern hemisphere, and which is not particularly demanding against the growth conditions. On average, bark as a forestry waste accounts for up to 12 % of the total wood-based biomass. Bark is unique, with a very different chemical composition depending on different factors such as tree species, growing regions, soil conditions and environmental impact, etc. [1]. The chemical composition is mainly wood extractives. Each tree species is characterized by a unique class of compounds, for example, oaks are characterized by a high content of tannins, while alders are characterized by diarylheptanoids and tannins, pine with stilbene, terpenoids and other compounds, etc. All of these compounds are plant secondary metabolites.

Parts of trees, in particular the tree bark, contain a wide range of plant secondary metabolites, which are rich sources of bioactive compounds with beneficial health effects [2,3]. Black alder bark has long been used worldwide in folk medicine due to its positive effects on human health [4,5]. Black alder bark contains various types of plant secondary metabolites, including terpenoids, flavonoids, diarylheptanoids, phenols, steroids, tannins and many others. The black alder bark has abundant diarylheptanoids containing the 1,7-diphenylheptane frame. Diarylheptanoids are the main effective components in the genus *Alnus* for their remarkable biological activities [6]. Phenolic compounds and flavonoids, which are widely found as secondary metabolites in *Alnus* plants, are important due to their ability to act as antioxidants [7,8,9,10]. Antioxidants of biological origin have been defined as “compounds that protect biological systems against the potentially harmful effects of processes or reactions that cause extensive oxidations”. Likewise, biologically active compounds from nature are increasingly used today to provide primary health protection [11,12]. In addition, these natural products serve as a basis for the invention and the production of new pharmaceutical products [13,14]. Synthetic antioxidants like BHT (butylated hydroxytoluene), TBHQ (tert-butylhydroquinone) and others are widely used worldwide due to the factors mentioned above. They are also much cheaper and chemically resistant, but in recent years researchers have shown the mutagenic and cancerogenic effects to human health of these synthetic antioxidants, and therefore it is necessary to look for alternatives to replace the synthetic antioxidants with natural antioxidants derived from plant-based raw materials [13,15]. The use of natural substances does not always provide the necessary effects because the needed active component content is low; therefore, it is necessary to find the optimal ways to separate and concentrate the biologically active compounds and to expand their use. One way is the extraction of natural substances, but generally the classical extraction methods are time-consuming, energy consuming and use toxic solvents; therefore, it is necessary to find new and effective solutions to separate biologically active extracts in safer, cheaper and more environmentally friendly manners [16]. The extraction of natural compounds from plant biomass depends on several factors, such as the extraction technique, the raw materials and the extraction solvents that are used [14]. One of the possible approaches is based on the concept of the biorefinery and the basic principles of ‘green’ chemistry, where all the parts of the biomass material are used effectively without any loss or unnecessary side stream products. Based on this approach, it is vital to develop new extraction processes that allow for the reduction of energy consumption, the use of alternative solvents, the use of renewable resources as feedstocks and, most importantly, allow for the obtainment of pure, high quality and highly biologically active extracts [17]. This work focuses on optimizing the extraction processes by changing the main extraction parameters, using alternative green solvents (water) to replace the organic solvents. The main goal of this extraction process development is to obtain high quality extracts with green techniques and to characterize them, determining the total content of polyphenols (TPC) and proanthocyanidins (PAC) in the extracts, which are principally responsible for the biological activity of the extracts, and to characterize the antioxidant activity of these extracts and to find the correlations between these parameters.

## 2. Results and Discussion

**Figure 1:**
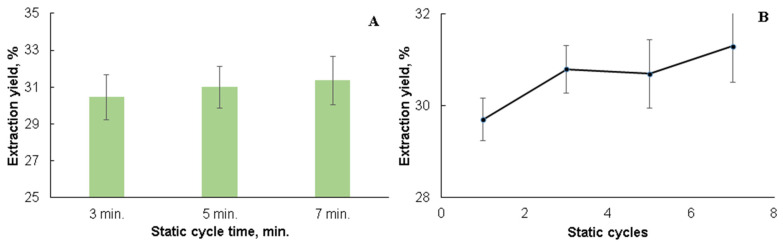
(**A**) The effect of the static cycle time on the total extraction yield% of black alder bark at 90 °C with solvents of increasing polarity. (**B**) The effect of the number of static cycles on the total extraction yield% of black alder bark extraction with solvents of increasing polarity at 90 °C.

The first step of the extraction method development was the optimization of each of the condition variables. Thus, the time of one static cycle in the sequential extraction with organic solvents of increasing polarity was changed from 3 to 7 min (three static cycles at 90 °C), as shown in Figure 1A. As is shown, using 5 min as a static cycle time is optimal for the extraction processes compared to 7 min, where the extraction yield is not significantly higher, because it is also necessary to take into account the nonhomogeneity of the biomass, which may slightly alter the extraction yield. After determining the best static cycle time (5 min), the number of static cycles was studied at 90 °C, thus changing from one to seven when determining the optimal conditions (Figure 1B) [18]. The optimal number of static cycles was selected as three, since, when comparing to five and seven cycles, the obtained yields did not increase so sharply as to outweigh the increased consumption of energy resources.

**Figure 2:**
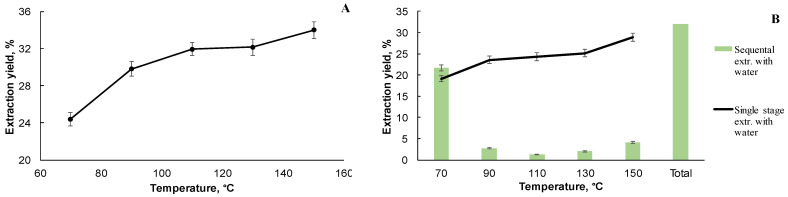
(**A**) Total extraction yield% of black alder bark sequential extraction with organic solvents, depending on extraction temperature at precustomized extraction parameters. (**B**) Total extraction yield% of black alder bark sequential and single stage extraction with deionized water, depending on extraction temperature at precustomized extraction parameters.

The significant increase of the extract yields in the results of seven repeated static cycles vs. that of three and five cycles was not established. Therefore, the extraction regimes with three static cycles were chosen for performing the next extraction experiments using the selected extraction solvents. In the further research of the extraction process with organic solvents with increasing polarity, deionized water and water with different pH, with single-step and sequential extraction, the extraction temperature was changed by 20 °C, from 70 °C to 150 °C (see Figure 2A).

As shown in Figure 2A, when increasing the temperature from 70 °C to 150 °C, the total yield of the extractives increases from 24 to 34% o.d.w. On the basis of these results, the optimal parameters for water extraction were selected: three static cycles, cycle time of 5 min with a single-step extraction at increasing temperatures. The extraction temperature was changed by 20 °C, from 70 to 150 °C (Figure 2B). As is shown, the same trend may be observed by using a single-stage extraction with deionized water, as in the case of black alder bark extraction with organic solvents, and by increasing the temperature from 70 °C to 150 °C, the yield of the extractives increases from 19% to 29%. A sequential extraction with deionized water at a temperature range of 70 °C to 150 °C (Figure 2B) shows that the highest yield of the extractives is directly obtained by the first extraction at a temperature of 70 °C, representing a yield of 22% from the dry bark. At a temperature up to 110 °C, the yield of the extractives decreases, but at a temperature above 130 °C, the yield of the extractives starts to grow slightly, which could be explained by the degradation and the subsequent solubilization of the biomass’ main macromolecular components, such as hemicellulose or lignin fragments, into extracts. Klarić, M., et al. [5] showed that the yield of the aqueous extract during the maceration procedure is only 4.3%, whereas with the extraction of the accelerated solvent extraction at 90 °C, an average of 23% of the extract was obtained. The accelerated solvent extraction method in this work shows the advantages of being able to obtain biologically active compounds from other plant materials of different origins according to the same principle, because, for example, with other classical extraction methods such as Soxhlet extraction it is not possible to work with solvent mixtures, but 40% (v:v) ethanol is used in this work. It should also be mentioned that the extraction is performed in excess of the boiling point of the solvents used, which is also not possible with classical extraction methods such as Soxhlet extraction, Batch extraction, etc.

**Figure 3:**
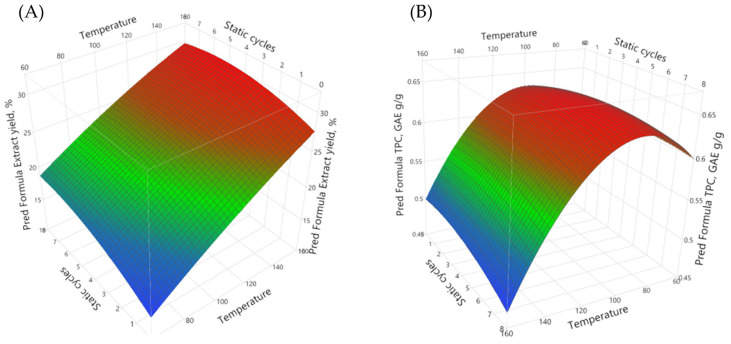
Surface plots of extraction yield (**A**) and TPC content in the extract (**B**) dependence of extraction conditions.

To obtain the optimal conditions of the accelerated solvent extraction, and taking into account the separated extract yield, the total phenolic content (TPC) in the extract and the specific energy consumption (Wh/g), the response surface methodology approach was used (see Figure 3). The obtained equations were used to produce the surface plots that show the extract yield and the TPC content in the separated extracts, including the dependency on the extraction conditions such as temperature, the static cycle count and the static cycle length.

Process optimization gives the maximal extraction yield and the TPC content in these extracts, while the minimal specific energy consumption can be achieved with a process condition where the temperature is 93 °C, the static cycle amount is set to three and the length of one static cycle is set at 5.3 min. Such conditions were also used in further experiments.

To make sure that water was a suitable solvent comparable with organic solvents to obtain valuable phytochemicals from the black alder bark, the extractions were performed with waters of various origins. Tap water was selected, as well as waters for which the acidity was changed during the electrolysis process with different pH, as well as water to which sodium carbonate was added so to make it alkaline. After the extraction, the yield of the extracts, the total polyphenols (TPC), the total proanthocyanidins (PAC) and the antioxidant activity (DPPH^•^) were determined (see Table 1).

Evaluating the obtained results according to the data of Table 1, it can be seen that the obtained results with different waters are close. The statistical analysis was done by performing the Kruskal–Wallis test (*n* = 4). For all the parameters, *p* values were >0.05, indicating no significant statistical difference between the used solvent systems. As for the extract with the deionized water, although the extraction yield is very close to tap water, it should be taken into account that for tap water, inorganic compounds in the form of salts and oxides increase the extraction yield. The same can be stated for the electrolytic waters and the alkaline water with the added sodium carbonate. These inorganic compounds from the used water entering the extract could potentially interfere with the further use of the separated extracts. Therefore, for ease of use, the deionized water was chosen for further experiments. Comparing the results with the works of other authors, for example, oak extracts obtained with water, the TPC varies in the range from 0.3–0.4 GAE g/g, while the water extracts from black alder bark contain more TPC, 0.56–0.63 GAE g/g [19], and the obtained values of antioxidant activity are also high compared with the extracts obtained from the different plants and their different parts of plants [20]. The methanol extract from pistachios shows a high TPC of 0.56 GAE g/g with a relatively high antioxidant activity, but it should be noted that it is obtained with a volatile organic solvent extraction agent that is not environmentally friendly [21]. As shown by Charu et al. [22], from Hungarian black alder bark using different solvents the authors have obtained extracts with much lower results in terms of the total polyphenol content, 21.25 and 15.14 GAE mg/g in 80% ethanol water and in 80% acetone water extracts, respectively. Comparing the results obtained in our work, which are on average 560 GAE mg/g and obtained with water as a solvent, the results are much higher. Similar results can be observed in the literature [23], where black alder bark samples were collected in the Kaliningrad region and extracted with water, and the results are on average 10 times lower for TPC.

After the extraction with deionized water, the residue was extracted sequentially with organic solvents to determine how much organic-soluble compounds in the selected organic solvents could be obtained with deionized water (see Table 2). The sequential extraction with the organic solvents at 90 °C was used as a reference.

**Figure 4:**
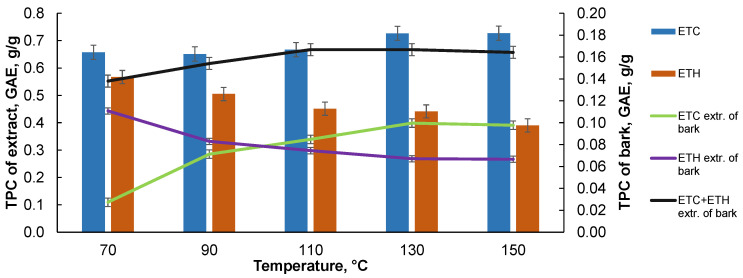
The total polyphenolic content (TPC) in the extracts isolated from black alder bark in the results of its sequential extraction with ETC and ETH and expressed as GAE g/g on the isolated extracts and the starting bark basis. (Data is shown as mean (*n* = 4) values ± standard deviation. Kruskal–Wallis test, *p* < 0.05.)

As can be seen from Table 2 with n-hexane, after the extraction with deionized water 1.77 ± 0.04%, the n-hexane extract is obtained, and the calculation shows that only 27.80 ± 0.81% of the lipophilic extracts are transferred in deionized water, while the hydrophilic extracts are transferred almost completely, with results being 85.68 ± 2.37 % and 91.60 ± 2.19% for the ethanol and ethyl acetate fractions, respectively. In order to assess the viability of the obtained extracts as valuable phytochemical compounds, the total polyphenol content (TPC) was determined for the extracts and expressed in GAE g/g. Black alder bark accelerated the solvent extraction results with organic solvents—hexane, ethyl acetate (ETC) and 40% (v:v) ethanol (ETH)—and the content of the polyphenols varies between the solvent used and depends heavily on temperature (see Figure 4).

**Figure 5:**
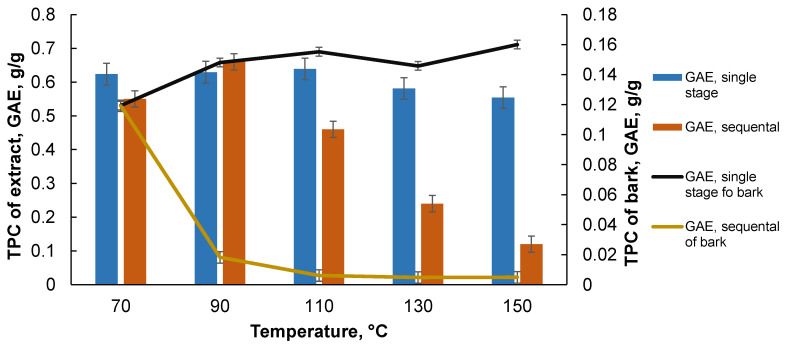
Black alder bark accelerated solvent extraction (single step and sequential) with deionized water total polyphenol content in the extracts and the bark depend on the temperature at predefined extraction parameters. Data is shown as mean (*n* = 4) values ± standard deviation. Kruskal–Wallis test, *p* < 0.05.

As can be seen, the higher content of polyphenols in all cases is found in the extracts obtained with the ETC as a solvent. As the extraction temperature increases, the content of the polyphenols in the ETH extracts decreases; on the other hand, it increases in the ETC extracts, which may be explained by the degradation of the high-molecular compounds and the easier transition in the ETC extraction. The ETC extraction was carried out prior to the ETH extraction. When recalculating the polyphenol content to the black alder bark biomass, it can be seen that an increased temperature reduces the total phenolic content in the ETH extracts but increases it in the ETC extract. The cumulative total polyphenol content of the extracts separated from the bark increases until a temperature of 110 °C, and then remains constant as the temperature is increased further. The extraction of the bark with the deionized water at different temperatures, in both the single-stage and sequential extractions, indicates that the content of polyphenols in the single-stage extraction, up to 110 °C, is almost constant and decreases slightly with the increase of temperature above 110 °C (Figure 5).

**Figure 6:**
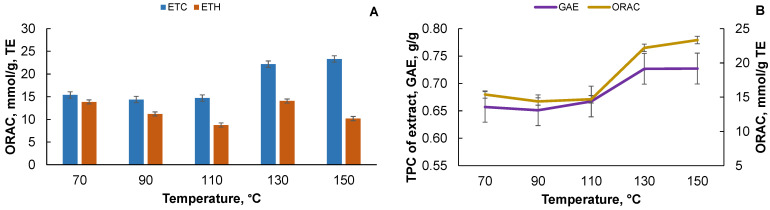
(**A**) Antioxidant activity (ORAC test) of the black alder bark ETC and ETH extracts and (**B**) the correlation between the antioxidant activity (ORAC test) of ETC extracts of black alder bark and total polyphenol (TPC) content. Data is shown as mean (*n* = 4) values ± standard deviation. For (**A**) Kruskal–Wallis test, *p* < 0.05.

For the sequential extraction with deionized water, the total polyphenol content decreases drastically as the yield of the extractives decreases (see Figure 2B). In the case of sequential extraction, this is explained by the transition of the polyphenolic compounds in the solvent during the first extraction step. However, in the case of single-stage extraction at a temperature above 110 °C, the polyphenol content is reduced, but not very drastically, and this could be explained by the ongoing physical-chemical processes in the extractable biomass, such as the degradation and subsequent solubilization of various macromolecules. When recalculating the results to the starting biomass, it can be seen that the polyphenol content increases with the single-stage extraction. It can also be concluded that the amount of polyphenol present in the obtained extracts in the case of the accelerated solvent extraction with deionized water, calculated on the bark, is comparable to the extracts obtained with the organic solvents, and on average is 0.16 GAE g/g on the bark. The values obtained for the antioxidant activity are compared with the predefined parameters, such as the total polyphenol content, to evaluate the correlation between them. Figure 6A shows a comparison of the oxygen radical antioxidant capacity (ORAC) test values for the black alder bark extracts obtained with the organic solvents, and the ORAC values are higher for the ETC extracts (the higher the ORAC value, the stronger the antioxidant) than for the ETH extracts, and this ratio increases with increasing temperature, as with Figure 5 where the ratio content of the polyphenols increases due to the temperature.

**Figure 7:**
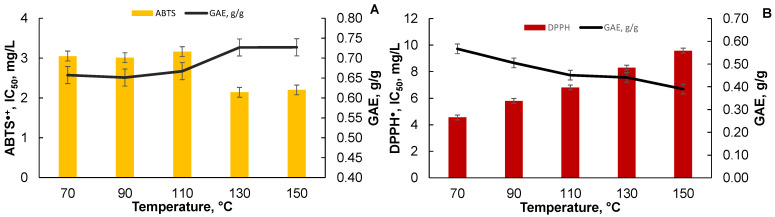
(**A**) Correlation of antioxidant activity (ABTS^●+^ test) of the black alder bark ETC extracts and total polyphenol content. (**B**) Correlation of antioxidant activity (DPPH^●^ test) of the black alder bark ETH extracts and total polyphenol content.

A positive correlation is observed for the ETC extracts of the black alder bark between parameters such as the ORAC test and the content of the total polyphenols GAE g/g (see Figure 6B), and the higher the antioxidant activity the extract contains, the more the total polyphenol compounds, and vice versa. Figure 7A shows the correlation between the total polyphenol content and the ABTS^●+^ test of the black alder bark ETC extract (in the ABTS^●+^ test, the lower the value, the higher the antioxidant activity). Figure 7B shows the correlation between the total polyphenol content and the DPPH^●^ test of black alder bark ETH extract. In both cases, the higher TPC content leads to higher antioxidant activity.

**Figure 8:**
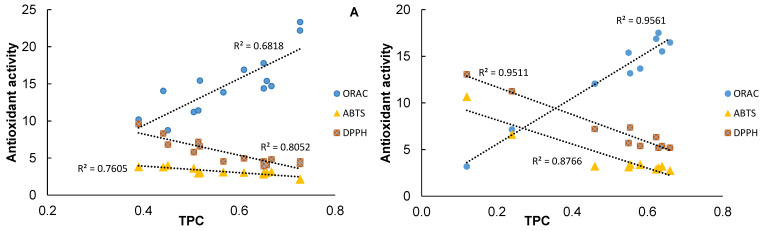
Correlations between antioxidant activity tests and total polyphenol content. (**A**) Sequential extraction with organic solvents at different temperatures (**B**) with one-step and sequential extraction with deionized water at different temperatures.

For the sequential water extraction of the black alder bark, there is also a positive correlation between the antioxidant activity in the ORAC, ABTS^●+^ and DPPH^●^ tests and the total polyphenol content in the extracts. Evaluating the correlation of the total antioxidant activity with the total content of polyphenols in the extracts obtained by sequential extraction with organic solvents at different temperatures (Figure 8A), and with one-step deionized water and sequential extraction at different temperatures (Figure 8B), shows that all the antioxidant activity test values are similar in all the tests, depending on the extraction parameters.

The values of Pearson’s correlation coefficients calculated for the relationship between the antioxidant activity and the total phenolic content was significantly higher than the critical values, which are equal to 0.532 and 0.638 for the extraction with organic solvents (N = 14) and water (N = 10), respectively.

**Figure 9:**
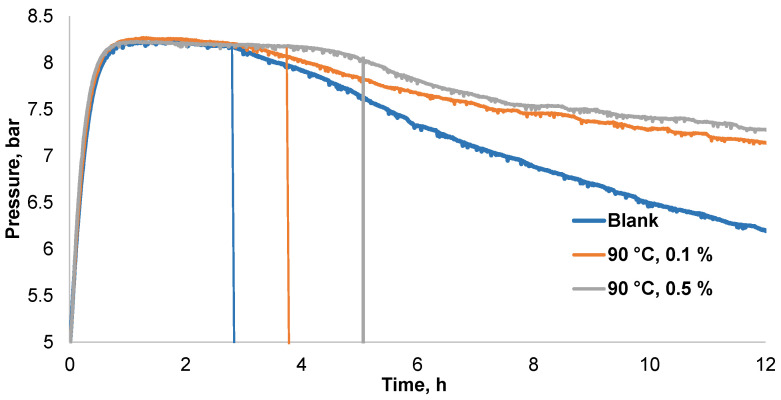
Effect of black alder bark deionized water extract oxidative stability of commercial mayonnaise at 120 °C demonstrated a higher PF than commercial mayonnaise without additive (blank).

Lipid oxidation is the main reason for the development of rancidity in fat-containing foods, particularly when they are rich in polyunsaturated fatty acids. Therefore, the main function of antioxidants is to extend the shelf time of foods by delaying lipid oxidation. It is obvious that the addition of 1.00–5.00 mg/g of black alder bark deionized water extract to mayonnaise significantly improves its oxidation stability in a dose-dependent manner. For instance, 1.00 and 5 mg/g of the extract increased the oxidation induction period (IP) from 2.32 h to 3.26 and 4.78 h, respectively (Table 3 and Figure 9).

**Figure 10:**
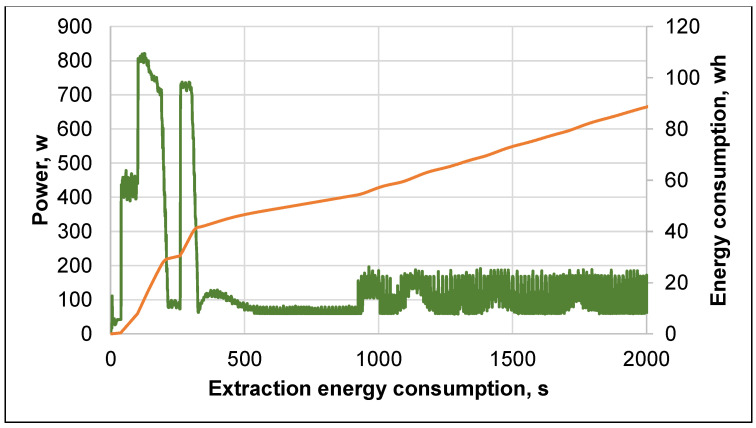
Measurement of energy consumption at 70 °C temperature for black alder bark deionized water extraction.

During the extraction of the black alder bark with deionized water at different temperatures, electrical power consumption measurements were performed. As shown in Figure 10, the largest energy consumption step is up to 400 s, which is explained by the heating of the accelerated solvent extraction furnace, which consumes nearly half of the energy capacity, where afterwards significantly less power is consumed in the extraction process.

These data were used as a basis for calculating the energy consumption to produce one unit of the extract at specified temperatures (see Table 4). The table shows that the energy consumption per unit of the extract does not change at temperatures of 130 °C and 150 °C, but up to 130 °C, it increases.

## 3. Materials and Methods

### 3.1. Plant Material

The raw material black alder (*A. glutinosa*) bark was harvested in May 2019 from 27-year-old trees grown in the Ogres municipality in Latvia. Growing conditions: Broad-leaved forest, moist soil and good growing conditions. After collection, the bark was dried at room temperature until the moisture content was approximately 10%, and then was milled using a Retch AS100 mill (Retch GmbH, Haan, Germany) at 1500 rpms with a 2 mm sieve. Milled bark was stored in a freezer at −18 °C until further analysis.

### 3.2. Chemicals and Reagents

N-hexane, ethyl acetate, 2,2-diphenyl-1-picrylhydrazyl (DPPH^•^), 2,2′-azino-bis (3-ethylbenzothiazoline-6-sulfonic acid) (ABTS^•+^), Folin–Ciocalteu reagent, gallic acid, methanol, 2,2′-Azobis (2-methylpropionamidine) dihydrochloride (AAPH), fluorescein and phosphate-buffered saline pH = 7.4 (PBS) were purchased from Sigma-Aldrich (Steinheim, Germany). All chemicals used for the analyses were of analytical grade, and all the test solutions were freshly prepared prior to use.

### 3.3. Sample Preparation

Bark was extracted with selected solvents (in a ratio of 1:3.5 (black alder bark:solvent)), deionized water, tap water, tap water after electrolysis with different pH (pH = 2.57 and pH = 11.62), deionized water with Na_2_CO_3_ and organic solvents: n-hexane, ethyl acetate and ethanol:water (40:60, v:v) in different sequences:

1. Only with organic solvents increasing their polarity, n-hexane→ethyl acetate→ethanol: water according to the previously developed methodology described in [11];
2. Only with deionized water: tap water and water with different pH;
3. Combined extraction methods: with deionized water and then with organic solvents sequential.

Changing extraction static cycle time 1–10 min at different temperatures (room to 150 °C) and changing number of static cycles (1–7). All the extraction procedures were done in triplicate at least. After the extraction, the used solvents were evaporated in vacuo (rotary evaporator, Heidolph Instruments) and freeze-dried (freeze dryer, Heto PowerDry PL3000), and the extracts obtained were stored at −20 °C. All results were expressed on a dry weight and ash-free basis.

### 3.4. Total Polyphenol Content (TPC)

The content of the extractable polyphenolics (TPC) in the obtained extracts were determined by Folin–Ciocalteu analysis [24]. An amount of 1 mL of hydrophilic extractives solution (in 50 % (v:v) ethanol) was added to 0.5 mL of the Folin–Ciocalteu phenol reagent followed by gentle shaking. After 5 min, 1 mL of 20 % (*w/v*) sodium carbonate was added. The solution was immediately diluted up to 5 mL with distilled water and mixed thoroughly. After 10 min, an optical density at 765 nm of the resulting blue complex was measured using a PerkinElmer Lambda 650 UV/VIS spectrophotometer against the blank, using gallic acid as the standard. The total phenolic contents were expressed as g of gallic acid equivalents (GAE) per g of dried extract sample [25].

### 3.5. Antioxidant Activity

The antioxidant activity of the obtained black alder bark extracts was assessed using the test with free radicals, DPPH^•^ and ABTS^•+,^, and were measured spectrophotometrically, where the free radical scavenging activity was expressed as the IC_50_ (the concentration required for 50 % inhibition of the free radical). The lower the IC_50_ value, the higher is the antioxidant activity. In the DPPH^•^ radical scavenging assay, the method described above was adopted with minor modifications [26]. A sample solution in DMSO (0.03 mL) was mixed for 15 min with 3.0 mL of a 1 × 10^−4^ mol/L DPPH^•^ in methanol solution, and then the absorbance at 517 nm of the mixture was immediately measured using a Perkin Elmer Lambda 625 UV/VIS spectrometer. ABTS^•+^ was produced by reacting 2,2-azino-bis (3-ethylbenzothiazoline-6-sulphonic acid) (ABTS) with potassium persulfate (K_2_S_2_O_8_). The absorbance at 734 nm was read at ambient temperature after 10 min. PBS solution was measured as a blank sample.

The kinetic ORAC test applied characterized the antioxidant potential in preventing fluorescein oxidation [27]. The final ORAC values were calculated using a regression equation (Y = aX + b) correlating the Trolox concentration (50–100 µM in PBS) and the net area under the fluorescein decay curve. The extracts’ radical absorbance capacity was expressed in this test as the Trolox equivalent (TE), the mmol per gram of the antioxidant sample. The higher TE value corresponds to the higher antioxidant activity.

The antioxidant activity of black alder bark deionized water extracts at two concentrations towards lipids oxidation in oxygen atmosphere was determined in a commercial mayonnaise in an Oxipres apparatus (Mikrolab Aarhus, Denmark) operating at 120 °C. The induction period (IP, in hours) of the oxidation was read at the time when the pressure began to decrease abruptly. The antioxidant activity of additives was assessed by their protection factors (PF):

PF = IPx/IPk(1)

where IPx is the oxidation IP with additive and IPk is the IP for sample without additive.

### 3.6. Total Proanthocyanidin Content

The acid-butanol method for determining the total proanthocyanidin content in the extracts was performed as described in the literature [28]. In a screw cap tube was added 1 mL of sample, 6 mL of acid-butanol reagent (950 mL n-butanol with 50 mL conc. HCl) and 0.2 mL of 2% Ferric ammonium sulfate solution in 2 mol HCl. The samples were vortexed and incubated for 50 min in a 90 °C degree water bath. Afterwards, the samples were cooled, and the absorbance was measured at 550 nm. Concentrations of proanthocyanidins were determined by the calibration curve of B2 epicatechin dimer standard [29].

### 3.7. Energy Consumption Measurements

The total energy consumption, depending on the temperature value and extraction duration, was measured using the electric power indicator SENECA S604E-6-MOD (SENECA, Padova, Italy). The specific energy consumption was calculated by taking into account the dry weight of the isolated extracts. Each experiment was repeated three times and the data were averaged.

### 3.8. Statistical Treatment of the Results

The measured values are shown as an average with a confidence interval (at a level of significance α = 0.05). Each measurement was performed at least in triplicate. Statistical calculations were carried out using MS Excel and IBM SPSS Statistics 21.0 (Microsoft Corp, Redmond, WA, USA).

## 4. Conclusions

By sequential extraction with organic solvents, it is possible to divide the extractable compounds from the black alder bark into groups to separate the lipophilic compounds with n-hexane, semipolar compounds with ethyl acetate and polar compounds with ethanol. Comparing the combined extracts obtained with the organic solvents, slightly more extractives are obtained compared to the water one-stage or the sequential extraction at different temperatures. As the temperature increases, the sequential extraction with the organic solvents and the single-stage extraction with the deionized water increases the yields of the extractives while the total content of polyphenols in the extracts varies with temperature. With the organic solvents, it can be observed that the TPC increases in the ethyl acetate extracts but decreases in the ETH extracts with increasing temperature. The TPC increases up to 110 °C degrees with the water extraction and then begins to decrease slightly with increasing temperature, while the TPC decreases dramatically with sequential water extractions, which is explained by the extraction yield. Comparing the extracts obtained from the sequential extraction of an accelerated solvent, the extracts with organic solvents (sum of ETC and ETH) and the extracts with the water contain comparable amounts of polyphenolic compounds converted to raw bark and form an average of 0.16 GAE g/g, which allows for the use of water as an alternative green solvent. However, in some cases the total polyphenol content of the individual extracts obtained with the ETC is as high as 0.8 GAE g/g. The extraction with water also allows us to obtain extracts with a good antioxidant activity when compared to the organic solvents, but when evaluating the physiochemical properties and costs of the solvents, water can be used as a green alternative to organic solvents to obtain extracts with a good potential to be natural antioxidants and therefore to prospectively compete in many areas with widely used synthetic antioxidants. It has been shown that by introducing the extracts obtained in the work with greener production methods in real food systems, it is possible to increase their stability by two times, and thus the high potential value of these extracts as antioxidants has also been shown.

## Figures and Tables

**Table 1:** Extraction yield, TPC, PAC and DPPH^•^ of the extracts obtained during the single-step extraction at predefined extraction parameters.

Used Solvent	Yield, %	TPC GAE g/g	PAC %	DPPH^•^ IC_50_ mg/L
Tap water *	23.31 ± 0.44	0.56 ± 0.012	13.42 ± 0.23	6.79 ± 0.15
Deionized water with Na_2_CO_3_, pH = 10.92	22.20 ± 0.39	0.56 ± 0.014	14.25 ± 0.25	7.12 ± 0.18
Electrolyzed water, pH = 11.62	22.87 ± 0.36	0.56 ± 0.014	14.28 ± 0.37	7.17 ± 0.14
Electrolyzed water, pH = 2.57	22.13 ± 0.32	0.58 ± 0.018	11.21 ± 0.28	6.26 ± 0.10
Deionized, water	23.53 ± 0.41	0.63 ± 0.02	21.59 ± 0.42	5.18 ± 0.11
Kruskal–Wallis test				
*p* value	0.332	0.1902	0.2164	0.2137

Data is shown as mean (*n* = 4) values ± standard deviation. * The supplementary file contains information on (water) annual averages, which are monitored in accordance with local legislation.

**Table 2:** Reference extraction yield with organic solvents in comparison with the sequential extraction with organic solvents after the single-step extraction with deionized water at predefined extraction parameters and organic soluble part in deionized water, %.

Solvent	Reference Yield, %	After Deionized Water Extraction, %	Organic Soluble Part in Deionized Water, %
n-hexane	2.45 ± 0.05	1.77 ± 0.04	27.80 ± 0.81
Ethyl acetate	10.95 ± 0.24	0.92 ± 0.017	91.60 ± 2.19
40% (v:v) ethanol	16.41 ± 0.36	2.35 ± 0.06	85.68 ± 2.37

Data is shown as mean (*n* = 4) values ± standard deviation.

**Table 3:** Black alder bark accelerated solvent extraction with deionized water comparative effect on the stability of mayonnaise.

Additive	Concentration of Additive, *w/w*, mg/g	Induction Period (IP), h	Protection Factor (PF)
Blank	-	2.32	1.00
Water extract	1.00	3.26	1.41
Water extract	5.00	4.78	2.06

**Table 4:** Black alder bark accelerated solvent extraction with deionized water energy consumption.

Temperature, °C	Total Energy Consumption, Wh	Specific Energy Consumption, Wh/g	Total Extraction Time, s
70	97.99 ± 2.54	13.21 ± 0.27	2315
90	131.19 ± 3.15	14.96 ± 0.36	2379
110	160.77 ± 4.66	16.96 ± 0.39	2556
130	207.33 ± 4.35	20.79 ± 0.54	2631
150	238.52 ± 6.21	20.51 ± 0.48	2697

Data is shown as mean (*n* = 4) values ± standard deviation.

## Data Availability

Not applicable.

## Supplementary Materials

The following are available online at https://www.mdpi.com/article/10.3390/plants10112531/s1.

## References

[1] Aspé E., Fernández K. (2011). Comparison of phenolic extracts obtained of Pinus radiata bark from pulp and paper industry and sawmill industry. Maderas. Cienc. y Tecnol..

[2] Malfa G.A., Tomasello B., Acquaviva R., Mantia A.L., Pappalardo F., Ragusa M., Renis M., Di Giacomo C. (2020). The Antioxidant Activities of Betula etnensis Rafin. Ethanolic Extract Exert Protective and Anti-diabetic Effects on Streptozotocin-Induced Diabetes in Rats. Antioxidants.

[3] Malfa G.A., Tomasello B., Acquaviva R., Genovese C., La Mantia A., Cammarata F.P., Ragusa M., Renis M., Di Giacomo C. (2019). Betula etnensis Raf. (Betulaceae) Extract Induced HO-1 Expression and Ferroptosis Cell Death in Human Colon Cancer Cells. Int. J. Mol. Sci..

[4] Vidaković V., Novaković M., Popović Z., Janković M., Matić R., Tešević V., Bojović S. (2018). Significance of diarylheptanoids for chemotaxonomical distinguishing between Alnus glutinosa and Alnus incana. Holzforschung.

[5] Klaric M., Oven P., Gorišek Ž., Španic N., Pervan S. (2016). Yield of Stirred Cold Maceration and Extraction of Milled European Black Alder Wood and Bark using Different Solvents. BioResources.

[6] Novaković M., Stanković M., Vučković I., Todorović N., Trifunović S., Tešević V., Vajs V., Milosavljević S. (2013). Diarylheptanoids from Alnus glutinosa Bark and Their Chemoprotective Effect on Human Lymphocytes DNA. Planta Med..

[7] Azman N.A.M., Skowyra M., Muhammad K., Gallego M.G., Almajano M.P. (2017). Evaluation of the antioxidant activity of Betula pendula leaves extract and its effects on model foods. Pharm. Biol..

[8] Lee S., Oh D.-G., Singh D., Lee H.J., Kim G.R., Lee S., Lee J.S., Lee C.H. (2019). Untargeted Metabolomics Toward Systematic Characterization of Antioxidant Compounds in Betulaceae Family Plant Extracts. Metabolites.

[9] Sati S., Sati O., Sati N. (2011). Bioactive constituents and medicinal importance of genus Alnus. Pharmacogn. Rev..

[10] Ren X., He T., Chang Y., Zhao Y., Chen X., Bai S., Wang L., Shen M., She G. (2017). The Genus Alnus, A Comprehensive Outline of Its Chemical Constituents and Biological Activities. Molecules.

[11] Mihaylova D., Desseva I., Stoyanova M., Petkova N., Terzyiska M., Lante A. (2021). Impact of In Vitro Gastrointestinal Digestion on the Bioaccessibility of Phytochemical Compounds from Eight Fruit Juices. Molecules.

[12] Gil-Chávez G.J., Villa J.A., Ayala-Zavala J.F., Heredia J.B., Sepulveda D., Yahia E.M., González-Aguilar G.A. (2013). Technologies for Extraction and Production of Bioactive Compounds to be Used as Nutraceuticals and Food Ingredients: An Overview. Compr. Rev. Food Sci. Food Saf..

[13] Soquetta M.B., Stefanello F.S., Huerta K.D.M., Monteiro S.S., da Rosa C.S., Terra N.N. (2016). Characterization of physiochemical and microbiological properties, and bioactive compounds, of flour made from the skin and bagasse of kiwi fruit ( Actinidia deliciosa ). Food Chem..

[14] Soquetta M.B., Terra L.D.M., Bastos C.P. (2018). Green technologies for the extraction of bioactive compounds in fruits and vegetables. CyTA J. Food.

[15] Bennett L.E., Jegasothy H., Konczak I., Frank D., Sudharmarajan S., Clingeleffer P.R. (2011). Total polyphenolics and anti-oxidant properties of selected dried fruits and relationships to drying conditions. J. Funct. Foods.

[16] ZY J., LR H. (2003). Effects of solvent and temperature on pressurized liquid extraction of anthocyanins and total phenolics from dried red grape skin. J. Agric. Food Chem..

[17] F C., MA V., G C. (2012). Green extraction of natural products: Concept and principles. Int. J. Mol. Sci..

[18] Lauberts M., Telysheva G., Venskutonis P.R., Lauberte L., Dizhbite T., Kazernavičiūte R., Pukalskas A. Diarylheptanoid-rich extract of grey and black alder barks: An effective dietary antioxidant in mayonnaise. Chem. Pap..

[19] Dudonne S., Vitrac X., Coutiere P., Woillez M., Mérillon J.M. (2009). Comparative study of antioxidant properties and total phenolic content of 30 plant extracts of industrial interest using DPPH, ABTS, FRAP, SOD, and ORAC assays. J. Agric. Food Chem..

[20] Wojdyło A., Oszmiański J., Czemerys R. (2007). Antioxidant activity and phenolic compounds in 32 selected herbs. Food Chem..

[21] Benmahieddine A., Belyagoubi-Benhammou N., Belyagoubi L., El Zerey-Belaskri A., Gismondi A., Di Marco G., Canini A., Bechlaghem N., Atik Bekkara F., Djebli N. (2021). Influence of plant and environment parameters on phytochemical composition and biological properties of Pistacia atlantica Desf. Biochem. Syst. Ecol..

[22] Agarwal C., Hofmann T., Visi-Rajczi E., Pásztory Z. (2021). Low-frequency, green sonoextraction of antioxidants from tree barks of Hungarian woodlands for potential food applications. Chem. Eng. Process. Process Intensif..

[23] Skrypnik L., Grigorev N., Michailov D., Antipina M., Danilova M., Pungin A. (2019). Comparative study on radical scavenging activity and phenolic compounds content in water bark extracts of alder (*Alnus glutinosa* (L.) Gaertn.), oak (*Quercus robur* L.) and pine (*Pinus sylvestris* L.). Eur. J. Wood Wood Prod..

[24] Singleton V.L., Orthofer R., Lamuela-Raventós R.M. (1999). [14] Analysis of total phenols and other oxidation substrates and antioxidants by means of folin-ciocalteu reagent. Methods Enzymol..

[25] Blainski A., Lopes G.C., de Mello J.C.P. (2013). Application and Analysis of the Folin Ciocalteu Method for the Determination of the Total Phenolic Content from *Limonium brasiliense* L.. Molecules.

[26] Dizhbite T., Telysheva G., Jurkjane V., Viesturs U. (2004). Characterization of the radical scavenging activity of lignins—Natural antioxidants. Bioresour. Technol..

[27] Prior R.L., Wu X., Schaich K. (2005). Standardized Methods for the Determination of Antioxidant Capacity and Phenolics in Foods and Dietary Supplements. J. Agric. Food Chem..

[28] Vieito C., Fernandes É., Velho M.V., Pires P. (2018). The effect of different solvents on extraction yield, total phenolic content and antioxidant activity of extracts from pine bark (*Pinus pinaster* subsp. *atlantica*). Chem. Eng. Trans..

[29] Schofield P., Mbugua D.M., Pell A.N. (2001). Analysis of condensed tannins: A review. Anim. Feed Sci. Technol..

