# Supplementary material for: Antioxidant Activity of Different Extracts from Black Alder (Alnus glutinosa) Bark with Greener Extraction Alternative
Source: Plants (Basel). 2021 Nov 21;10(11):2531. doi: 10.3390/plants10112531 (PMC8621986; doi:10.3390/plants10112531)
Supplement: Supplementary file 1 [file plants-10-02531-s001.zip › plants-1460555-supplementary.pdf]

Average numerical value of the parameters in 2020 from Riga water.

Aluminum: 0.02 mg/L

Ammonium: 0.02 mg/L

Antimony: <1.25 µg/L

Arsenic: <1 µg/L

Boron: 0.5 mg/L

Cyanides: 2 µg/L

Water hardness: 1.8 mmol/L

Turbidity: 0.2 NDV

Iron: 0.08 mg/L

Mercury: <0.1 µg/L

Electrical conductivity: 548 µS/cm (20°C)

Fluorides: 0.13 mg/L

Chlorides: 89 mg/L

Chrome: <0.5 µg/L

Cadmium: <0.1 µg/L

Manganese: 0.02 mg/L

Sodium: 37 mg/L

Nickel: 1.2 µg/L

Nitrates: 1.9 mg/L

Nitrites: 0.004 mg/L

Chemical oxygen demand: ($KMnO_4$) 2.4 mg/L; O_2

pH: 7.5 pH

Selenium: <1 µg/L

Sulphates: 38 mg/L

Lead: 1.1 µg/L

Copper: 0.08 mg/L
